#  Effects of Cisplatin and its Analogues on the Permeability of Human Erythrocyte Membrane

**DOI:** 10.1155/MBD.1995.73

**Published:** 1995

**Authors:** J.-F. Lu, K. Wang, X.-Z. Sun, F. Xing, P.-D. An, Z.-H. Yang, J.-J. Yin

**Affiliations:** 1 The National Laboratory of Natural and Biomimetic Drugs Beijing Medical University Beijing 100083 China; 2 National Biomedical ESR Center Medical College of Wisconsin Milwaukee Wl 53226 USA

## Abstract

The biomembrane is postulated as the initial target when Platinum(II) complexes attack cells. In this work, a spin-labeling ESR technique has been used to study the effects of cis-DCDP, cis-DBDP, cis-DIDP, trans-DCDP, and cis-DADP on the permeability of human erythrocyte membrane. We monitored the reduction processes of the ESR signal of a nitroxide spin label, (TEMPO), which leaks out through the membrane and is reduced by the external ascorbate. Our results indicate that cisplatin and its analogues can enhance the permeability of membranes to small moieties such as TEMPO and ascorbate, and the differences between these compounds are related to features of the leaving group. In addition, changes in the order parameter of 5DS spin label in membrane indicate that hydrolysis of these Pt(II) complexes result in membrane damage.


### EFFECTS OF CISPLATIN AND ITS ANALOGUES ON THE PERMEABILITY OF HUMAN ERYTHROCYTE MEMBRANE

J.-F. Lu[1], K. Wang[1], X.-Z. Sun[1], F. Xing[1], P.-D. An[1], Z.-H. Yang[1] and J.-J. Yin*[2]

[1] The National Laboratory of Natural and Biomimetic Drugs, Beijing Medical University, Beijing 100083, China
[2] National Biomedical ESR Center, Medical College of Wisconsin, Milwaukee, WI 53226, USA

### ABSTRACT

The biomembrane is postulated as the initial target when Platinum(II) complexes attack cells. In this work, a spin-labeling ESR technique has been used to study the effects of cis-DCDP, cis-DBDP, cis-DIDP, trans-DCDP, and cis-DADP on the permeability of human erythrocyte membrane. We monitored the reduction processes of the ESR signal of a nitroxide spin label, (TEMPO), which leaks out through the membrane and is reduced by the external ascorbate. Our results indicate that cisplatin and its analogues can enhance the permeability of membranes to small moieties such as TEMPO and ascorbate, and the differences between these compounds are related to features of the leaving group. In addition, changes in the order parameter of 5DS spin label in membrane indicate that hydrolysis of these Pt(II) complexes result in membrane damage.

### INTRODUCTION

Cisplatin is a broadly used anticancer agent (1). However, its usage has been limited by kidney toxicity and nephrotoxicity. In the past several years, numerous studies were focused on the mechanism of the interaction between cisplatin and cells, with particular attention to the interaction with DNA (1, 2). Recently, the research has been extended to membrane lipid, membranae protein (3) and cytoskeleton (4). It is believed that the drug-membrane interaction plays a key role in the toxicity of antitumor agents. A "multi-targets" mode has been suggested to describe the cellular response as a whole with the biomembrane as the initial target (3). Any exogenous compounds, including drug molecules must penetrate the membrane before arriving at other intracellular targets. The interaction of drug molecules with membrane lipid and membrane protein will influence membrane permeability. This process is thought to be the basis of toxicity and activity of drugs. In addition to cis-DCDP, several other analog compounds such as carboplatin (CBDCA) have been used clinically (5).

In the present work, the effects of cis-DCDP and its analogues on membrane penetration are reported. Electron spin resonance spectroscopy (6) has been used to investigate the reaction kinetics between ascorbate and the paramagnetic group of a spin label intercalated in membrane. The effects of various Pt(II) complexes, such as cisdichlorodiammineplatinum (cis-DCDP) (I), cisdibromodiammineplatinum (cis-DBDP) (II), cisdiiododiammineplatinum (cis-DIDP) (III), transdichlorodiammineplatinum (trans-DCDP) (IV) and cisdiamminediaquoplatinum (cis-DADP)(V) on human erythrocyte

*Current address: Instrumentation and Biophysics Branch, U.S. Food and Drug Administration, HFS 717, 200 C Street, Washington, DC 20204.

membrane permeability for the ascorbate ion or the non-ionic spin label TEMPO molecule have been investigated comparatively.

An additional experiment was conducted to study the influence of Pt(II) complexes on lipid organization in human erythrocyte membrane. This was done by measuring theand order parameter of the stearic acid spin label, which is located on the headgroup of the bilayer.

### MATERIALS AND METHODS

Pt(II) complexes were obtained from the Institute of Pharmacy, Beijing Medical University. The n-doxyl stearic acid spin labels containing $^{14}N$ nitroxide moieties at the 5, 7, 12 and 16 positions (5DS, 7DS, 12DS and 16DS respectively) and the spin label 2,2,6,6-tetramethylpiperidine-1-oxyl (TEMPO) were purchased from Sigma Chemical Co. (St. Louis, MO). Ascorbic acid was from Beijing Phamacia (Beijing). All chemicals were analytical grade.

**Sample preparation and ESR measurement**. Fresh human blood was obtained from the Beijing Red Cross Blood Supply Station. The biomembrane was prepared according to the Dodge method (7). 100 ml of red cells (2 x $10^8$ /ml) was broken in 4000 ml of PBS buffer (pH = 8.0, 5.0 mmol/L) at 4°C for 2 hours and then centrifugally washed three times with PBS buffer at 15,000 rpm, and at 4°C for 10 min. A PBS buffer (pH 7.4, 50 mmol/L) was used for the final centrifugation step. The white deposit was collected as the erythrocyte membrane and stored at -20°C for use. The membrane protein concentration is 2.5 mg/ml.

A modified Sanberg method (8) was used to prepare samples. The mole concentration ratio of membrane to spin label is 100:1. The mixture of membrane and spin label was incubated at 4°C overnight. Before ESR measurement, the samples were washed centrifugally until there was no free spin label in the supernatant. Saline solutions of various Pt(II) complexes were added to the spin-labeled membranes to a final concentration of 1.5 mM. The control samples, with saline solution only, were incubated 15 min at 25°C, pH 7.2. Conventional ESR experiments were performed on a Bruker ESP-300 X-band ESR spectrometer equipped with ER-4111 variable temperature accessory. Spectra were obtained with 2 mW incident microwave power and 100 KHz field modulation of 2G.

**Kinetics of spin label reduction study**. Before ESR measurement, at 0°C, a freshly prepared ascorbate solution was added with ten-fold excess molar concentration into the samples. Time accounting began immediately after mixing. The sample was packed in a capillary and mounted into the resonance cavity. The ESR spectrum was recorded every 2 minutes while automatically stepping the central magnetic field by 1 Gauss after each record. The paramagnetic nitroxide group of spin label was reduced by ascorbate, which resulted in the reduction of the ESR signal amplitude (Fig 1).

The reduction rate of the spin label was quantified by comparing the signal amplitude on the low-field region of the ESR spectrum, which decreases as the measurement time increases. The half-time T, when the amplitude of ESR signal reduced to 50% of the original value, gives the rate of the solutes across the membrane, and the $T_{out}$ and $T_{in}$ represent the permeability of TEMPO molecule and ascorbate ion across the membrane bilayer, respectively. Also, $T_{in}$ was used for the stearic acid spin label studies.

The permeation rate of ascorbate into the membrane is temperature dependent (6).

Samples with TEMPO spin label were measured at 0°C, since at this temperature, ascorbate ion can not penetrate into biomembrane. However, with the stearic acid spin-labeled system, the experiments were carried out at room temperature at which the ascorbate ion can enter the membrane bilayer.

The effects of Pt(II) complexes on lipid organization of membrane were studied by measuring the order parameter of spin label 5DS. The order parameter S which indicates the fluidity of biomembrane, was calculated following ref.(9).

### RESULTS

1. The effects of Pt(II) complexes on the permeability of TEMPO spin labels from the lipid bilayer to the outside of the membranes.

Three-dimensional ESR spectra of TEMPO spin label in the human erythrocyte membrane at different times are shown in Fig. 1. The ESR signal amplitude is reduced by adding ascorbate ion at 0°C.

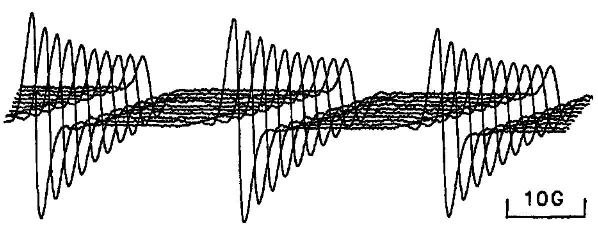

Fig. 1. Three-dimensional ESR spectra of TEMPO spin label in the human erythrocyte membrane reduced by adding ascorbate at O°C. The time interval was 2 minutes and the step change of the center magnetic field was 1G automatically (X-band, 2mW incident microwave power and 100 KHz field modulation of 2G).

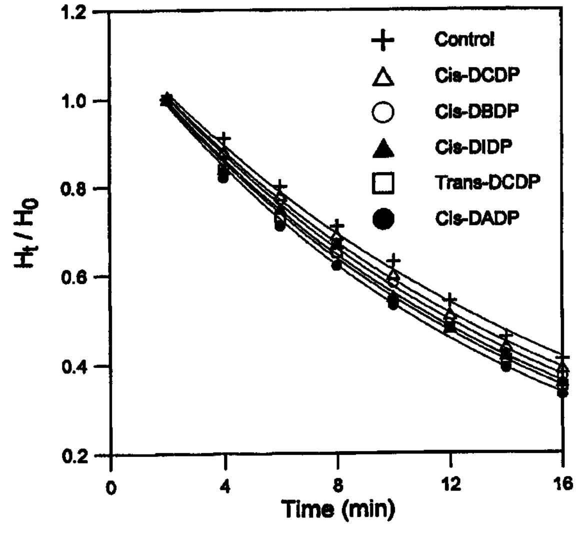

Fig. 2. Time dependence of the normalized decay curves of the signal amplitude ($H_t/H_o$) of TEMPO spin label in the human erythrocyte membrane reduced by ascorbate at O°C in the presence of various Pt(II) complexes. $H_o$ is the amplitude of ESR signal recorded at 2 minutes after the mixing samples.

The half-time $T_{out}$ was derived from the normalized decay curve (Fig. 2) and is given in Table 1. The value of the $T_{out}$ affected by various Pt(II) complexes is: $T_{out}(0) > T_{out}(I) > T_{out}(II) > T_{out}(III) > T_{out}(IV) > T_{out}(V)$, and $(T_{out})_{max}/(T_{out})_{min} = 1.28$.

2. At room temperature, ascorbate ion can penetrate into membrane. The permeation of ascorbate from extra-membrane medium into lipid bilayer was affected by various Pt(II) complexes. Figure 3 is the ESR spectrum of 5DS spin label in human erythrocyte membrane at different times, and shows that the amplitude of ESR signal was reduced by adding ascorbate. Half-time $T_{in}$ was derived from normalized decay curve (Fig. 4) and is given in Table 1. $T_{in}$ was affected by various Pt(II) complexes: $T_{in}(0) > T_{in}$ (I) $> T_{in}$ (II) $> T_{in}$(III) $> T_{in}$ (IV) $> T_{in}$(V), but $(T_{in})_{max}/(T_{in})_{min} = 1.81$. It was noted that at double the concentration of cis-DADP, the half-time $T_{in}$(V), increased dramatically up to 18.83 min.

Table 1. Half-time $T_{out}$ for spin label TEMPO leaking out through membrane and being reduced by ascorbate at 0°C, and half-time $T_{in}$ for ascorbate anion entering the membrane bilayer and interact with spin label 5DS in the lipid bilayer in the presence of Pt(II) complexes with a final concentration of 1.5 mM (n=5, and the standard deviation is ± 0.3).

| | Control (0) | cis-DCDP (I) | cis-DBDP (II) | cis-DIDP (III) | trans-DCDP (IV) | cis-DADP (V) |
|---|---|---|---|---|---|---|
| $T_{out}$ | 13.1 | 12.2 | 11.6 | 11.2 | 10.7 | 10.2 |
| $T_{in}$ | 15.95 | 14.1 | 12.7 | 12.1 | 10.3 | 8.8 |

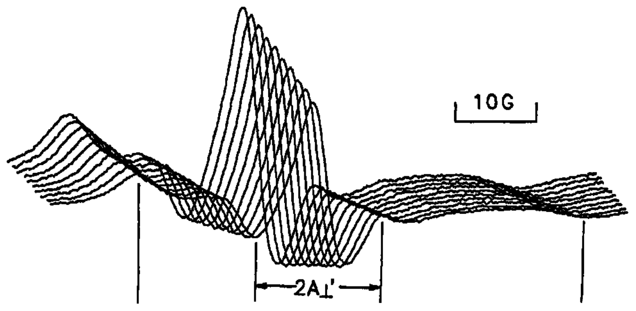

Fig. 3. Three-dimensional ESR spectra of 5DS spin label in the human erythrocyte membrane as a function of the time after adding ascorbate. The time interval was 2 minutes and the step change of center magnetic field was 1 Gauss automatically. Where 2A' and 2A⊥' are the parallel and perpendicular parts of the hyperfine splitting in the spectra.

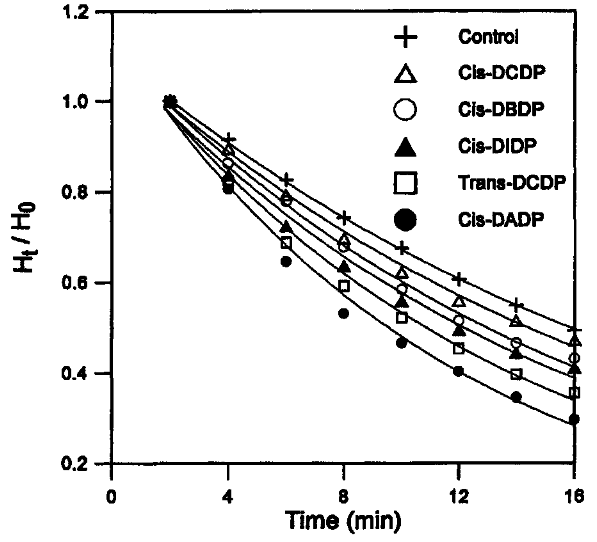

Fig. 4. Time dependence of the normalized decay curves of the signal amplitude ($H_t/H_0$) of 5DS spin label in human erythrocyte membrane reduced by ascorbate at room temperature in the presence of various Pt(II) complexes. $H_0$ is the amplitude recorded at 2 minutes after the mixing samples.

Table 2. Half time $T_{in}$ (min) of spin labels (nDS) at different depth of lipid bilayer of human erythrocyte membrane being reduced by ascorbic acid in the presence of Pt(II) complex with a final concentration of 1.5 mM (n=3).

| | control (0) | cis-DCDP (I) | trans-DCDP(IV) | cis-DADP(V) |
|---|---|---|---|---|
| 5DS | 14.08 ± 0.70 | 13.28 ± 0.54 | 12.10 ± 0.45 | 9.79 ± 0.44 |
| 7DS | 28.31 ± 1.27 | 25.20 ± 1.13 | 24.86 ± 1.02 | 22.33 ± 0.98 |
| 12DS | 66.81 ± 3.34 | 60.02 ± 2.41 | 77.10 ± 3.44 | 55.08 ± 2.31 |
| 16DS | 34.20 ± 1.39 | 25.29 ± 1.28 | 49.49 ± 2.02 | 24.81 ± 1.28 |

3. The half-time $T_{in}$ for various stearic acid spin labels in human erythrocyte membrane was affected by Pt(II) complexes. For spin labels 5DS, 7DS, 12DS and 16DS, the nitroxide moieties are located at different depths of the acyl chain of lipid bilayer. The ESR signal reduction is related to the permeation of ascorbate ion in the membrane. The permeability of ascorbate ion into the lipid bilayer is influenced by various Pt(II) complexes, and the data are listed in Table 2. The nitroxide group of the spin label is located near the hydrophilic region for 5DS and close to the hydrophobic region for 16DS in the membrane. Under the influence of Pt(II) complexes, the times at which ascorbate ion enters the membrane are: $T_{in}$(5DS) < $T_{in}$(7DS) < $T_{in}$(16DS) < $T_{in}$(12DS). The corresponding permeability of ascorbate ion with Pt(II) complexes were all higher than the controls.

4. The effects of various Pt(II) complexes on the membrane fluidity. Order parameter S of stearic acid spin label in the membrane is used to characterize the membrane fluidity. The S values of 5DS influenced by different Pt(II) complexes in human erythrocyte membrane are listed in Table 3.

Table 3. The order parameter S of 5DS spin label in the human erythrocyte in the presence of various Pt(II) complexes with a final concentration of 1.5 mM after incubation 1 hour.

| | control (0) | cis-DCDP (I) | cis-DBDP (II) | cis-DIDP (III) | trans-DCDP (IV) | cis-DADP (V) |
|---|---|---|---|---|---|---|
| S | 0.746 ±0.016 | 0.775 ± 0.014 | 0.778 ± 0.018 | 0.787 ± 0.020 | 0.787 ± 0.030 | 0.791 ± 0.035 |

### DISCUSSION

The method of spin labeling electron paramagnetic resonance was shown to be applicable to the study of the membrane permeability affected by exogenous compounds. It provides a unique tool to observe the permeation process of solutes across membranes or within membrane bilayer at the molecular level (10). The drug effects on the membrane are probable due to their influences on the surface structures of biomembranes and the interactions of Pt(II) complexes with membrane lipid and membrane protein. There is a consequent change in the membrane fluidity and the conformations of relevant molecules.

A transport process of solute from extracellular space includes at least two fundamental steps: first is the entry from the extracellular aqueous phase into the lipid medium (across membrane surface), and the second step is the diffusion along the acyl chains of phospholipid molecules (diffusion process). A small molecule, such as the spin label TEMPO, is likely to penetrate through the membrane according to a simple passive diffusion following a concentration gradient. However, the transport of ascorbate ions is perhaps due to a facilitated diffusion which has been mediated by a special ion pathway in the membrane.

The experimental results show that the permeability of human erythrocyte membrane increases in the presence of Pt(II) complexes for both TEMPO molecule and ascorbate ion. When Pt(II) complex attack membranes the electrostatic interactions could take place at the membrane surface, and the positively charged hydrolytic products of these Pt(II) complexes tend to alter the charge barrier of the membrane surface. Since the hydrolysis of Pt(II) complex is a slow and reversible process, the system is in a non-equilibrium during the experiment. Both hydrolytic and non-hydrolytic Pt(II) complexes exist outside the membrane and change the surface feature of the biomembrane in different manners, which then affect the permeability of different solutes (11).

The substitution ability of a ligand (or a leaving group) of a Pt(II) complex in an aqueous system was summarized (12) in the following order: $H_2O > Cl^- > Br^- > I^-$. However, the hydrolysis of dichlorodiammineplatinum(II) was limited by the relatively high concentration of chloride ions in the saline solution. Eventually, the two positive charges of Cisdiaqueodiammineplatinum(II) will influence the surface potential and alter the negatively-charged barrier of the membrane at some concentration that will facilitate solute transportation. We noted that the permeation rate of solutes was reduced or $T_{in}$ was increased when the cis-DADP concentration was doubled. Perhaps, this is induced by an increasing of the net positive charges in the polar region of the bilayer. The influence of hydrolysis is an important factor for ion transport. The changes in the permeation half-time $T_{in}$ for ascorbate is more sensitive than $T_{out}$ for TEMPO molecule in the presence of 1.5 mM Pt(II) complex. The former changed about 45% and the latter was 22% in this experiment; although, the change trends of $T_{in}$ and $T_{out}$ were similar. On the other hand, Pt(II) non-hydrolytic complexes are neutral, and their entrance into the lipid bilayer most dependent on the lipid/water partition coefficient. It has been found that the penetration rates of Pt(II) into the membrane are in turn increased for cis-DCDP, cis-DBDP and cis-DIDP in response to their lipid/water portioning (13).

Biomolecules, such as membrane lipids and membrane proteins, possess various motions and conformations, both influencing the transport of any external substance. The interactions between Pt(II) complexes, membrane lipids, and proteins will change the fluidity and the conformation of the membrane and damage the cell membrane at the same time. The two ammonia molecules of cis-DCDP interact with the phosphate groups of the polar heads of phospholipids and decrease the mobility of the polar heads in the liposome (14). Platinum can bind with a high affinity to sulfur-containing molecules, such as cysteine, methionine, glutathione, metallothionein and other proteins (2). The binding rate will be faster after hydrolysis of the Pt(II) complex. The results showed that the order parameters of membrane lipid at the polar head group were increased by 1.4, 1.8, 2.1, 3 and 3.5% in the presence of cis-DCDP, cis-DBDP, cis-DIDP, trans-DCDP and cis-DADP, respectively, as compared with the control. With the increasing of the order in the assembly of bilayer acyl chains, or order parameter, solutes diffuse more easily into the bilayer. Studies of the permeability at different depths of the membranae bilayer using different spin labels give a clear picture within the bilayer. They show Pt(II) complexes not only affect the membrane surface, but also interact with various membrane molecules enhancing solutes to transport through the membrane bilayer. In general, the longer the diffusion distance, the larger the half-time of the permeation. The data shows that $T_{in}$ (16DS) < $T_{in}$ (12DS) is very interesting. It is possible that the vertical fluctuation of the phospholipid is responsible for the results (15).

Transdichlorodiammine-platinum possesses a six times higher lipid/water partition coefficient than cisdichlorodiammine-platinum, easy hydrolysis, and strong interaction with membrane molecules; all of which promote solutes across membrane. It has been observed that the trans-Pt reacts more rapidly with GSH compared with cis-Pt(2). The lipid-solubility for cis-DIDP, cis-DBDP and cis-DCDP are lower in turn and are all smaller than trans-DCDP's.

In conclusion, cisplatin and its analogues interact with membranes and affect the biophysical property of the lipid bilayer. This work reveals that the effects of various platinum complexes with different ligands on the permeability of membrane are related to their hydrolyzing, partitioning, and interacting with membrane molecules. These effects are certainly important for the transportation of other ions, such as $Na^+$, $K^+$, $Cl^-$ and $Ca^{++}$ ions which maintain the normal physiological functions of cells and may contribute to the toxicity mechanism of Pt(II).

### ACKNOWLEDGEMENT

This work was supported by National Natural Science Foundation of China and the grant from the China-Cornell fellowship programs.

### REFERENCES

1. J. Reedijk (1987). *Pure & Appl. Chem.*, **59**(2), 181-192.
2. E. L. M. Lempers and J. Reedijk (1991). *Advances in Inorganic Chemistry*. **37**, 175-217.
3. K. Wang (1988). *Pure & Appl. Chem.*, **60**(8), 1279-1284.
4. X. Y. Su, J. J. Xiao and K. Wang (1990). *J. Mol. Sci.*, **6**(2), 110-116.
5. M. T. Cleare and J. D. Hoeschele. (1973). *Bioinorg. Chem.*, **2**, 187.
6. R. D. Kornberg and H. M. Mcconnell (1971). *Biochemistry*, **10**(7), 1111-1120.
7. J. T. Dodge, C. Mitchell and D. Hanahan (1963). *Arch, Biochem, Biophys.*, **100**, 119-130.
8. H. E. Sanbeg (1969). *Arch. Biochem. Biophys.*, **133**, 144-152.

9. B. J. Gaffinly (1975). *Proc. Nat. Acad. Sci.*, **72**(22), 664-668.
10. S. Schreier-Muccillo, D. Marsh and I. C. P. Smith (1976). *Arch. Biochem. Biophys*. **172**, 1-11.
11. R. Glaser (1990) in "*Biophysics in the cell surface*" (eds: R. Glaser and D. Gingell), Springer-Verlav New York, 173-187.
12. K. Wang (1989). *Bioinorganic Chemistry*, 225-237. Qinghua Univ, Press. Beijing.
13. M. W. Zhang, L. Z. Li, R. C. Li, P. Y. Dou and K. Wang (1993). *J. Chemical of Chinese Univ.*, **14**(10), 1351-1353.
14. Z. W. Shen, Z. P. Sun and N. M. Zhao, (1991). *Chinese Science Bulletin*, **36**(2), 149-153.
15. J.-J. Yin, J. B. Feix and J. H. Hyde (1990). *Biophysics J*, **58**, 713-720.

**Received: July 6, 1994 - Accepted: July 27, 1994 - Received in revised camera-ready format: October 14, 1994**

